# Exploring the pathogenesis of osteomyelitis accompanied by diabetic foot ulcers using microarray data analysis

**DOI:** 10.1097/MD.0000000000033962

**Published:** 2023-10-27

**Authors:** Pan Fan, Huanhuan Ye, Chenhua Zhu, Hu Xie

**Affiliations:** a Department of Orthopedics, The Second people’s Hospital of Yichang, China Three Gorges University, Yichang, China.

**Keywords:** DEGs, DFU, hub genes, osteomyelitis, PPI

## Abstract

Although numerous studies have shown distinctive similarities between osteomyelitis and diabetic foot ulcers (DFU), the common pathogenesis of both is not fully understood. The current research focuses on an in-depth study of the molecular and pathway mechanisms involved in the complication of these 2 diseases. We downloaded clinical information on osteomyelitis (GSE30119) and DFU (GSE29221) from the GEO database, along with gene expression matrices. Differentially expressed genes (DEGs) among normal individuals and patients with osteomyelitis; normal individuals and patients with DFU were identified by R software, and thus common DEGs were confirmed. We then analyzed these differential genes, including the functional pathway analysis, protein–protein interaction (PPI), modules and hub genes establishment, and transcription factor regulatory networks. We identified 109 common DEGs (46 up-regulated and 63 down-regulated genes) for subsequent analysis. The results of PPI network and the functional pathway analysis revealed the importance of immune response and inflammatory response in both diseases. Among them, chemokines and cytokines were found to be closely related to both osteomyelitis and DFU. In addition, the tumor necrosis factor (TNF) pathway and Staphylococcus aureus infection were found to have more significant roles too. The 12 most essential key genes were later screened by cytoHubba, including matrix metalloproteinases (MMP) 1, MMP3, MMP9, IL8, C-X-C chemokine receptor (CXCR) 2, C-X-C motif chemokine ligand (CXCL) 9, CXCL10, CXCL13, FCGR3B, IL1B, LCN2, S100A12. CXCL10, and MMP1 were validated using the least absolute shrinkage and selection operator (LASSO) and support vector machine-recursive feature elimination (SVM-RFE) algorithms. Osteomyelitis and DFU share similar molecular and pathway mechanisms. These common key genes and pathways may provide new directions toward the future study of osteomyelitis and DFU.

## 1. Introduction

It has been well researched that diabetic foot ulcers (DFU) can significantly increase the risk of osteomyelitis. Furthermore, patients with previous history of DFU are more likely to develop osteomyelitis than patients without DFU.^[1]^ Osteomyelitis and DFU both affect the inflammatory milieu, including inflammatory responses and immune responses, inflammatory chemokines and cytokines such as serum tumor necrosis factor (TNF)-α, interleukin (IL) 1β, and IL8.^[2]^ Although DFU are considered a risk factor for osteomyelitis, the common pathogenesis of these 2 diseases remains poorly studied. Neutrophils may play a vital role in this, and osteomyelitis and DFU may have a common pathogenesis.

Osteomyelitis is often associated with bone infections and is caused by Staphylococcus aureus, in which neutrophils play a key role in the inflammatory response.^[3]^ In patients with osteomyelitis, altered neutrophil Bax genes, reduced spontaneous apoptosis, and prolonged survival are correlated and may play a role in the pathogenesis of osteomyelitis.^[4]^ Neutrophils can store secreted tissue proteinase G (CTSG) in neutrophils, which can regulate inflammation and activate matrix metalloproteinase (MMPs).^[5]^ CTSG can enhance susceptibility to osteomyelitis. Cytokines and chemokines secreted by neutrophils are also closely correlated with the pathogenesis of osteomyelitis, and the risk of infection in osteomyelitis is greatly increased following a significant rise in IL1β expression.^[6]^ The cytokine IL-8 is a chemokine that binds with high affinity to C-X-C chemokine receptor (CXCR) 1 and CXCR2 receptors on neutrophils. In osteomyelitis, there is an upregulation of secretion of the neutrophil-attracting factor IL-8.^[7]^

In the early stages of diabetic foot ulcer patients, neutrophils enter the site of inflammation and participate in the earliest stages of the inflammatory response, playing a vital role in infection control and tissue debridement.^[8]^ Large numbers of neutrophils were found in the unhealed wounds of diabetic mice, and the depletion of neutrophils accelerated the closure of the wounds. Subsequently, there was a progressive decrease in neutrophils and pro-inflammatory cytokines IL-1B and IL6, whilst an increase in the cellular component chemokine ligand (CCL) 2, a well-known mediator of neovascularization.^[9]^ However, as the disease develops, the level of neutrophil chemokine IL-8 decreases, and neutrophils are unable to rebuild accordingly, thus reducing the release of chemokine CCL2 and affecting angiogenesis.^[10]^ In addition, neutrophil migration function is weakened, and bacteriostatic and bactericidal functions are significantly inhibited.^[11]^ Neutrophil function is also affected abnormally, producing large quantities of reactive oxygen species and proteases MMPs that can damage normal healthy tissues,^[12]^ and reducing the release of several growth factors that promote wound healing, such as VEGF, platelet-derived growth factor (PDGF), and transforming growth factor-β (TGF-β), delays wound repair.^[13]^

Transcriptomic analysis may help to explore novel pathogenesis of osteomyelitis in combination with DFU infections. This study was conducted to investigate the pivotal genes and related pathways shared among osteomyelitis and DFU. We downloaded 2 datasets (GSE30119 and GSE29221) containing clinical and genetic information from the GEO database, then utilized R software to identify common differentially expressed genes (DEGs) and their functions in osteomyelitis and DFU. The STRING database and Cytoscape software were used to construct protein–protein interaction (PPI) networks and identify critical gene modules and their key genes. We then identified 12 key pivotal genes and verified their expression levels. In addition, the transcription factor regulatory networks of these pivotal genes were further analyzed. Moreover, C-X-C motif chemokine ligand (CXCL)10 and MMP1 were identified as preferable diagnostic markers for osteomyelitis and DFU using machine learning algorithms.

In this study, the key genes and pathways common to osteomyelitis and DFU were identified, with aims to shed light on their common pathogenesis and provide potential treatment directions. To our knowledge, this is the first study to explore the gene signatures osteomyelitis and DFU using a systemic bioinformatic analysis approach.

## 2. Materials and methods

### 2.1. Data source

The clinical information and gene expression profiles of GSE30119 and GSE29221 were downloaded from the GEO (http://www.ncbi.nlm.nih.gov/geo/). Two microarray datasets (GSE30119 and 29221) were obtained from the GPL6947 platform, Illumina HumanHT-12 v3 Expression Bead Chip array. The GSE30119 contains 44 osteomyelitis tissues and 34 normal subjects (Normal). The GSE29221 consists of 12 DUF patients (DFU) and 12 healthy samples (Healthy) from the human skeletal muscle.

### 2.2. Identification of DEGs

All original data were normalized via the R software (version 4.0.2). Different gene expression analyses were completed by the “GEOquery” and “limma” packages. Packages were downloaded from the Bioconductor database (http://www.bioconductor.org/). Genes with | logFC (fold change) | >0.5 and *P* value <.05 were considered significant cutoffs to select DEGs.

### 2.3. Functional analyses of DEGs

Gene Ontology (GO) analysis is considered a vital tool for labeling genes, gene sequences, and products. The Kyoto Encyclopedia of Genes and Genomes (KEGG) consists of a series of databases on genomes, proteomics, biological pathways, drugs, diseases, and chemical substances. We used the online bioinformatics tool DAVID (https://david.ncifcrf.gov) to conduct the GO and KEGG functional analyses. *P* value <.05 was considered significant.

### 2.4. PPI network establishment and modules analyses

The STRING database (https://string.embl.de/) was used to generate the screened genes of the PPI networks, with the combined score >0.4 sets as the cutoff criterion. The Cytoscape (version 3.7.2) was used for visualizing the intersectional regulatory networks. The key gene modules were identified via the MCODE, the Cytoscape plugin, with degree cutoff = 2, K-core = 2, node cutoff = 0.2, and max depth = 100.

### 2.5. Identification and analysis of hub genes

Cytohubba, a Cytoscape plugin, was utilized to calculate the degree of proteins and identify the top 12 hub genes according to 3 common algorithms (Degree, Betweenness, Closeness). The co-expression network of 12 hub genes was established using GeneMANIA online tool (http://www. genemania.org/). The GeneMANIA was acknowledged as a dependable tool for recognizing internal associations for gene sets.

### 2.6. Verification of hub genes expression

GSE16129 and GSE7014 were used for validating the mRNA expression levels of selected hub genes. The GSE16129 dataset consists of 33 osteomyelitis patients and 10 normal samples. GSE7014 contains 20 DFU patients and 5 control samples. The contrast between the 2 groups of data was conducted via the *t* test. *P* value <.05 were considered a significant cutoff.

### 2.7. Prediction and validation of transcription factors (TFs)

Transcriptional Regulatory Relationships Unraveled by Sentence-based Text mining contained human target genes related to TFs and was used for the identification of transcriptional regulatory networks. TFs associated with hub genes were identified via the Transcriptional Regulatory Relationships Unraveled by Sentence-based Text mining database, and a *P* value <.05 were considered a significant cutoff. We then validated the expression of the TFs in GSE30119 and GSE29221 with the *t* test.

### 2.8. Validation of hub genes

The support vector machine-recursive feature elimination (SVM-RFE) is used to identify the optimal hub genes by dependent on the msvmRFE and e1071packages for SVM modeling. The least absolute shrinkage and selection operator (LASSO) regression analysis is utilized for evaluating the identified hub biomarkers combined with the expression data to find the best appropriate genes using the R package “glmnet.”

### 2.9. Ethical approval

The ethical approval was not necessary because no animal or human experiments were performed in this study.

## 3. Results

### 3.1. Identification of DEGs

**Figure 1.**
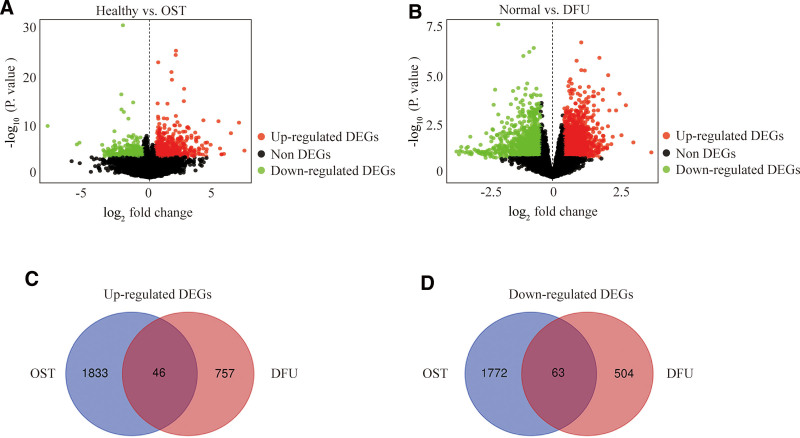
Volcano and Venn diagram. (A) The volcano map for DEGs in Healthy and osteomyelitis tissues of GSE30119. (B) The volcano map for DEGs in Normal and DFU tissues of GSE29221. (C and D) Venn plot of up-regulated and down-regulated DEGs in the 2 datasets. DEGs = differentially expressed genes, DFU = diabetic foot ulcers.

The respective DEGs (3714 in GSE30119 and 1370 in GSE29221) were identified using the R packages under normalizing the matrix information of GSE30119 and GSE29221 combined with the corresponding clinical information (Fig. 1A and B). 109 common DEGs were obtained, including 46 up-regulated genes and 63 down-regulated genes (Fig. 1C and D).

### 3.2. Analysis of the functional pathways of common DEGs

**Figure 2.**
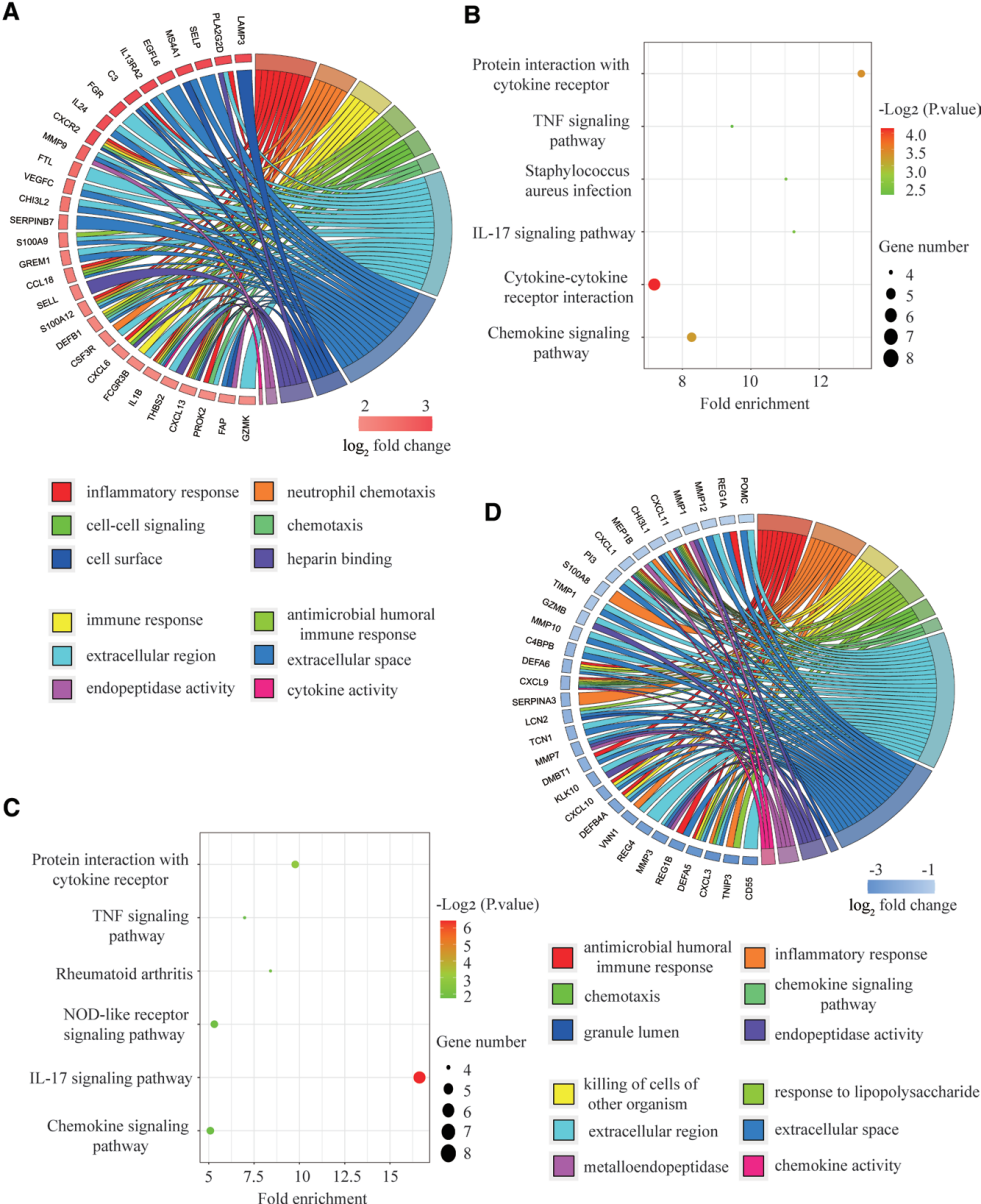
Functional analysis of the up-regulated and down-regulated genes GO and KEGG functional analysis (A and B) of the up-regulated genes. KEGG and GO functional analysis (C and D) of the down-regulated genes. GO = gene ontology, KEGG = Kyoto encyclopedia of genes and genomes.

GO and KEGG pathway enrichment analyses were performed on 46 up-regulated and 63 down-regulated genes to investigate their biological functions and pathways. For the up-regulated genes, we identified the 12 most significant pathways by GO analysis, among which the 4 most significant ones were neutrophil chemotaxis (*P* = 2.62E-10), inflammatory response (*P* = 6.46E-10), antimicrobial humoral immune response (*P* = 7.5E-10), and KEGG pathway, immune response (*P* = 7.84E-8), and immune response (*P* = 5.03E-105) (Fig. 2A). By KEGG analysis, we selected the 6 most significant pathways, of which the 4 most significant were Cytokine-cytokine receptor interaction (*P* = 7.22E-5), Protein interaction with cytokine receptor (*P* = 4.46E- 4), Chemokine signaling pathway (*P* = 5.90E-4), and Staphylococcus aureus infection (*P* = 5.01E-3). Notably, 3 genes (NCF2, RAC2, and MMP9) were involved in Staphylococcus aureus infection, the most prevalent etiology of osteomyelitis in diabetic foot ulcer infections (Fig. 2B).

For the down-regulated genes, the results of KEGG analysis demonstrated the IL-17 signaling pathway (*P* = 3.38E-7), Protein interaction with cytokine receptor (*P* = 1.14E-3), Chemokine signaling pathway (*P* = 1.49E-2), and TNF signaling pathway (*P* = 1.81E-2) were the 4 most significant pathways (Fig. 2C). The 4 most significant enrichment pathways identified by GO analysis were antimicrobial humoral immune response (*P* = 1.31E-14), inflammatory response (*P* = 2.72E-11), cellular response (*P* = 2.33E-8), and chemotaxis (*P* = 8.04E-8) (Fig. 2D).

These results suggest that cytokines and chemokines could influence the local immune microenvironment and thus be involved in the pathogenesis of both inflammatory diseases.

### 3.3. PPI network establishment and module analysis

**Figure 3.**
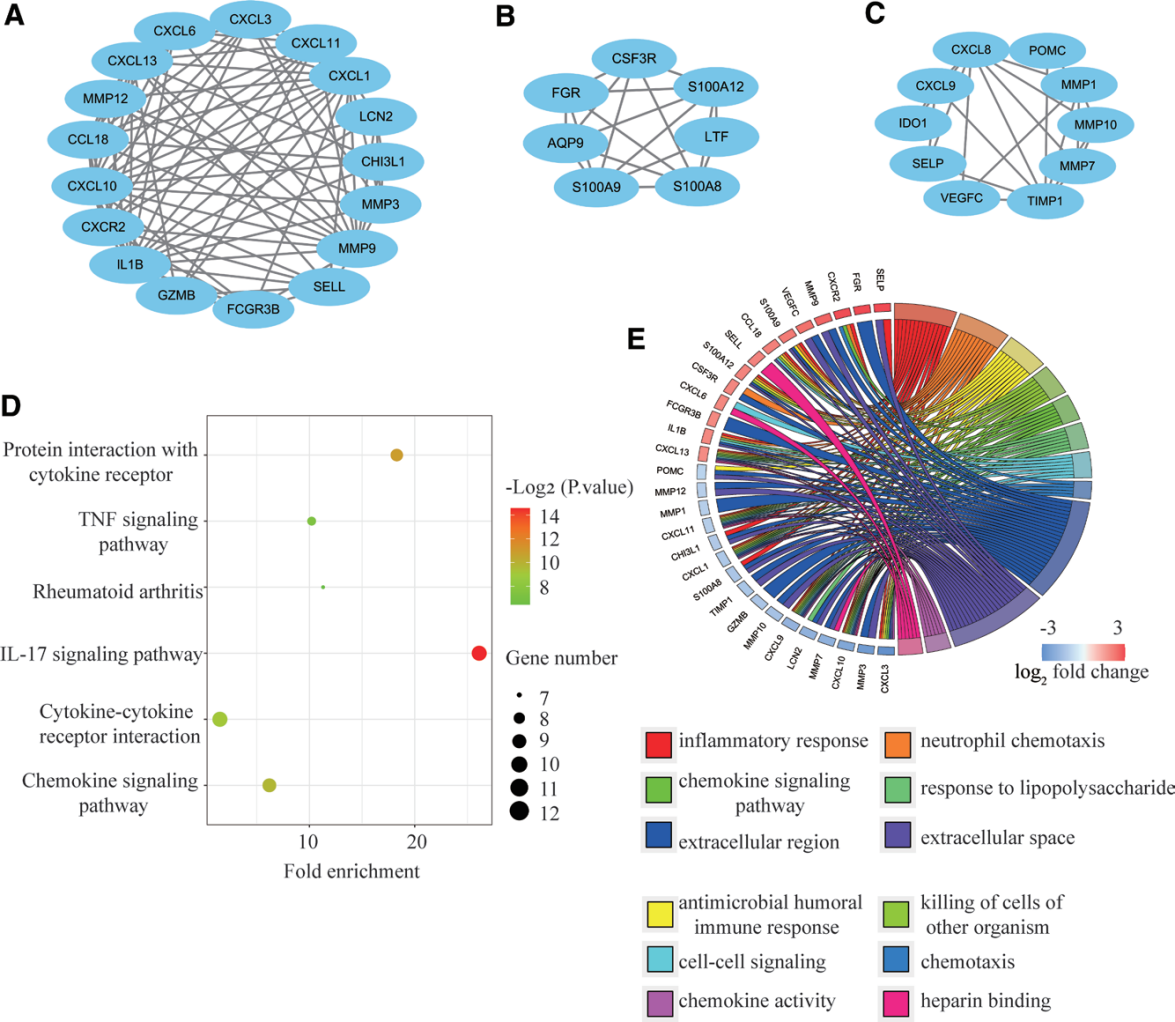
Significant gene clustering modules and functional analysis of the genes. (A–C) Three significant gene modules. (D and E) KEGG and GO functional analysis of the genes. GO = gene ontology, KEGG = Kyoto encyclopedia of genes and genomes.

The STRING online software was used to analyze the PPI network of 109 common DEGs. Cytoscape MCODE plugin was used to obtain key gene modules, resulting in 3 tightly linked modules, including 34 DEGs and 130 interaction pairs (Fig. 3A–C). KEGG functional analysis showed that these genes were mainly enriched in the IL-17 signaling pathway (*P* = 4.53E-15), Protein interaction with cytokine receptor (*P* = 2.35E-11), Chemokine signaling pathway (*P* = 3.55E-10), Cytokine-cytokine receptor interaction (*P* = 1.34E-9) (Fig. 3D). GO functional analysis showed that these genes are mainly involved in immune and inflammatory response-related pathways, including neutrophil chemotaxis (*P* = 1.45E-25), antimicrobial humoral immune response (*P* = 1.76E-19), inflammatory response (*P* = 1.16E-18), and cytokine-cytokine receptor interaction (*P* = 1.34E-19) (Fig. 3E).

### 3.4. Identification and analysis of hub genes

**Figure 4.**
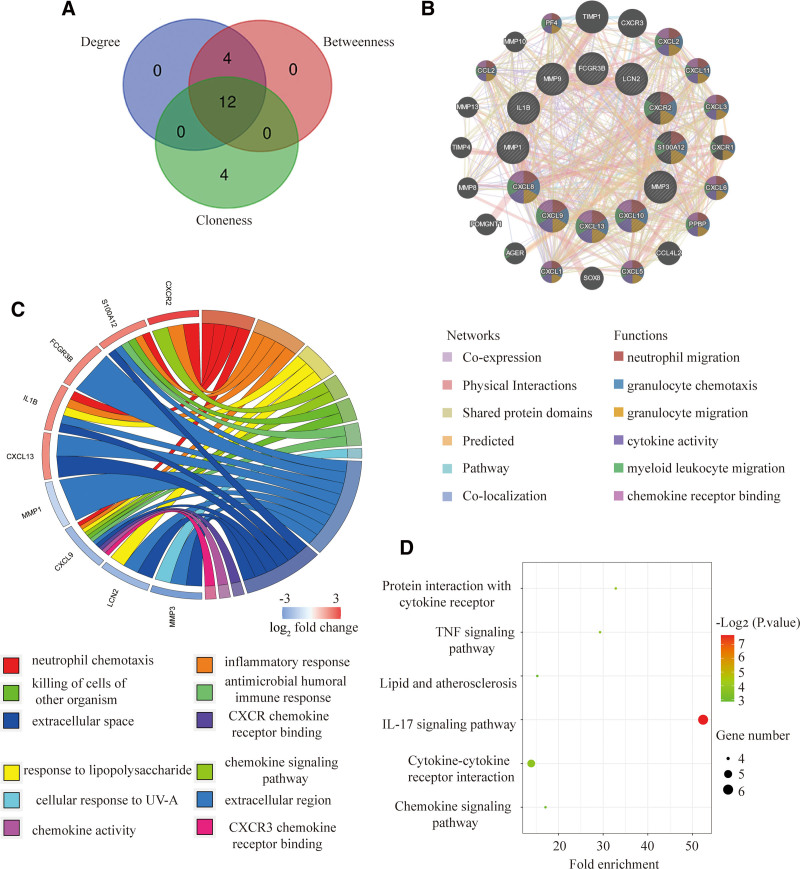
Co-expression network and functional analysis of hub genes. (A) The Venn plot of 12 overlapping hub genes screened out by 3 algorithms. (B)The diagram of 12 hub genes and their co-expression patterns via GeneMANIA. (C and D) GO and KEGG functional analysis of the hub genes. GO = gene ontology, KEGG = Kyoto encyclopedia of genes and genomes.

We obtained the top 16 hub genes using the 3 most classical algorithms of cytoHubba, and the intersection was taken using a VEEN graph. Of them, 12 were found to be common hub genes, including IL8, CXCL9, CXCL10, CXCL13, CXCR2, FCGR3B, IL1B, LCN2, MMP1, MMP3, MMP9, and S100A12 (Fig. 4A). We completed the analysis of the expression network and related functions of these genes, mainly closely related to the immune response, using the GeneMANIA database. The results demonstrate an elaborate PPI network with Co-expression of 51.44%, Physical Interactions of 21.86%, Shared protein domains of 14.57%, Predicted of 5.33%, Pathway of 0.99%, and Co-localization of 1.71% (Fig. 4B). GO functional analysis showed that these genes were enriched in neutrophil chemotaxis, inflammatory response, cellular response to lipopolysaccharide (LPS), chemokine- KEGG analysis showed that these genes were enriched in IL-17 signaling pathway, Cytokine-cytokine receptor interaction, and TNF signaling pathway (Fig. 4C). receptor interaction, TNF signaling pathway, Chemokine signaling pathway, and antimicrobial humoral immune response (Fig. 4D). These results highlight the crucial roles of chemokines, cytokines, antimicrobial responses, and LPS in both diseases.

### 3.5. Verification of hub genes expression

**Figure 5.**
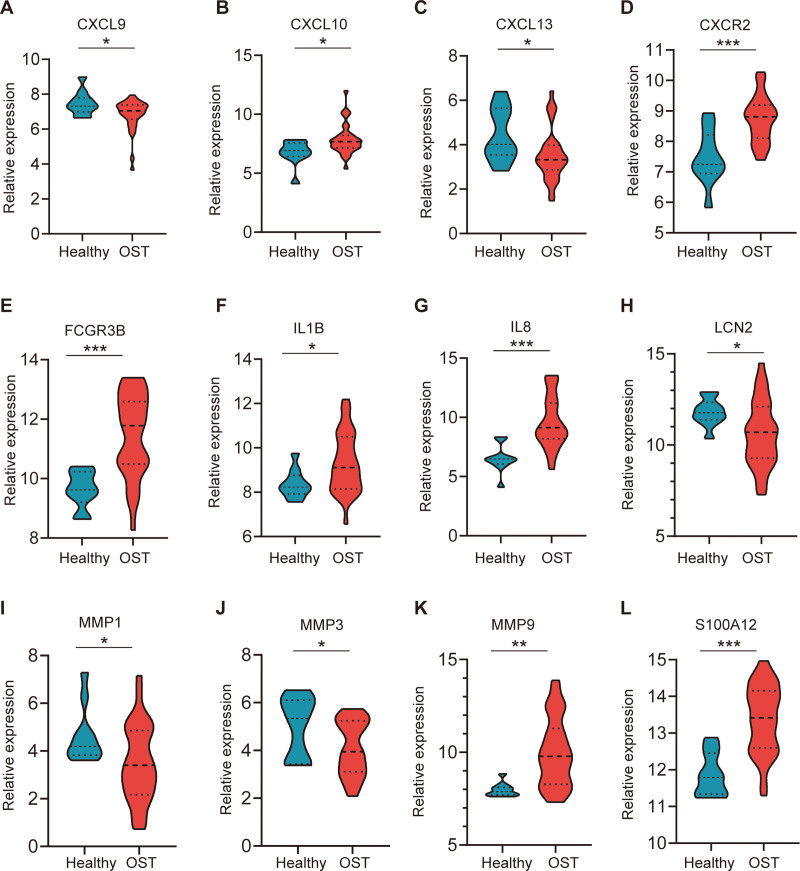
The different expression of hub genes in GSE16129. The difference between the 2 groups via the *t* test. OST, osteomyelitis. *P < .05; **P < .01; ***P < .001.

**Figure 6.**
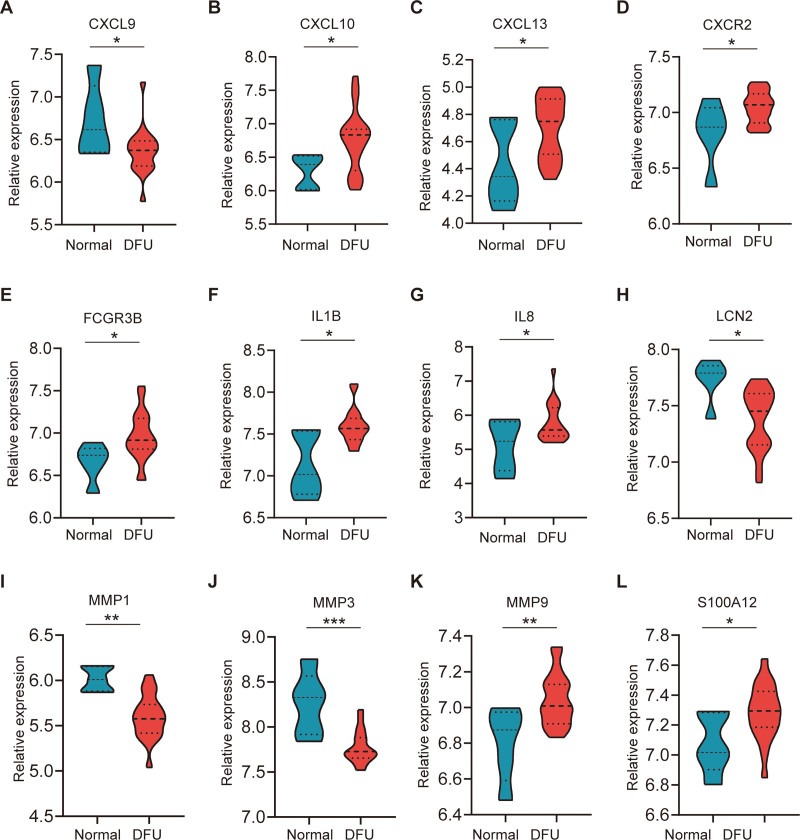
The different expression of hub genes in GSE7014. The difference between the 2 groups via the *t* test. DFU = diabetic foot ulcers. *P < .05; **P < .01; ***P < .001.

To confirm the reliability of the expression levels of these hub genes, we processed 2 additional datasets containing information related to osteomyelitis and DFU and analyzed the expression levels of these key genes. The results showed that patients with osteomyelitis had increased expression of CXCL10, CXCL13, CXCR2, FCGR3B, IL1B, IL8, MMP9, and S100A12 and decreased expression of CXCL9, LCN2, MMP1, and MMP3 compared to normal subjects (Fig. 5). Similarly, the changes in expression of all hub genes were consistent in patients with DFU (Fig. 6).

### 3.6. Prediction and validation of TFs

**Figure 7.**
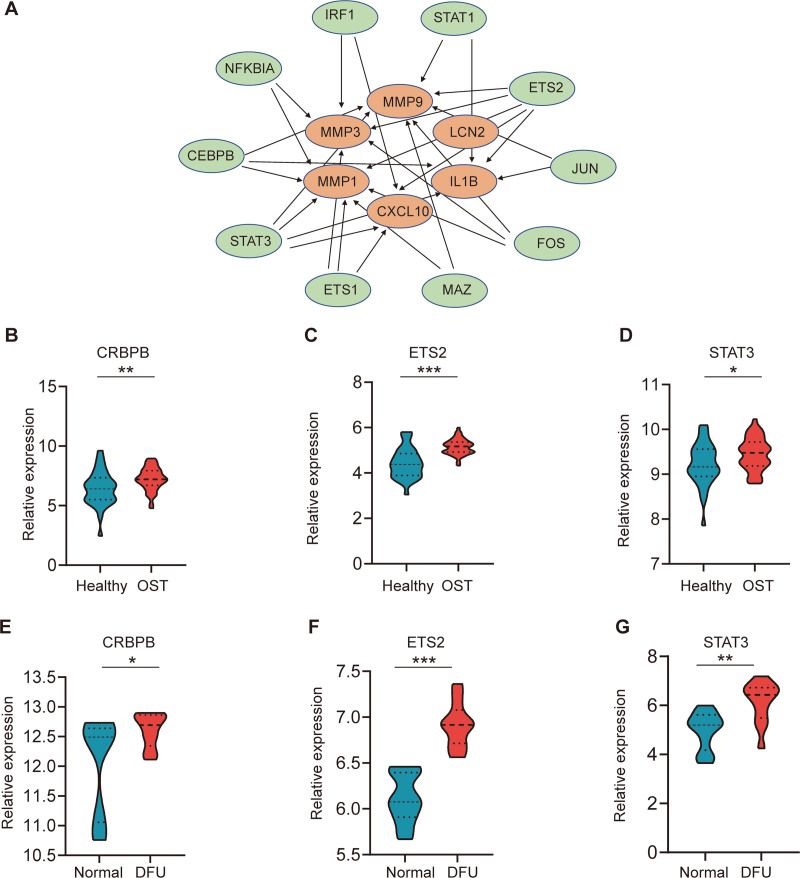
TFs regulatory network of hub genes and expression in GSE30119 and GSE29221. (A) TFs regulatory network of hub genes. TFs were filled in green, and the hub genes were filled in brown. (B and C) The different expressions of TFs in GSE30119 and GSE29221. *P < .05; **P < .01; ***P < .001. DFU = diabetic foot ulcers, OST = osteomyelitis, TFs = transcription factors.

We used the TRUST database to identify 10 TFs that may be involved in the regulation of these genes (Fig. 7A). We further found that 3 TFs, including CRBPB, ETS2, and STAT3 were highly expressed in both osteomyelitis and DFU (Fig. 7B–G). They synergistically regulated the expression of 4 hub genes (CXCL10, IL1B, MMP1, and MMP9).

### 3.7. Verification of potential shared diagnostic gene biomarkers according to the machine learning algorithms

**Figure 8.**
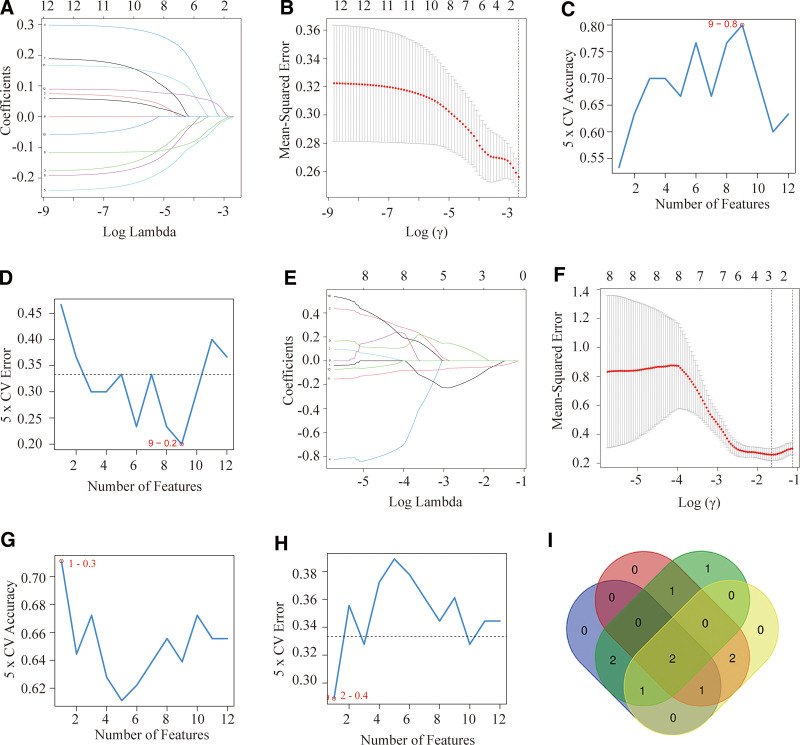
Two algorithms using for hub genes selection. (A and B) Ten-time cross-verification for tuning parameter identification and coefficient profiles of 12 hub genes in the LASSO model. (C and D) The accuracy and error of the estimate results for the SVM-RFE algorithm in the GSE30119. (E–H) The selective genes from LSAAO and SVM-RFE algorithm in the GSE29221. (I) Venn diagram of an overlap of 2 genes in modules between osteomyelitis and DFU. DFU = diabetic foot ulcers, LASSO = least absolute shrinkage and selection operator, SVM-RFE = support vector machine-recursive feature elimination.

Two machine learning algorithms, LASSO and SVM-RFE were used for selecting core biomarkers from 12 candidate hub genes produced by cytoHubba. We conducted the LASSO algorithm to select a set of 6 hub genes (Fig. 8A and B) and the SVM-RFE algorithm to identify a series of 6 genes (Fig. 8C and D) from GSE30119. In the GSE29221, LASSO and SVM-RFE algorithms were used to identify 6 and 7 genes, respectively (Fig. 8E–H). Finally, we identified CXCL10 and MMP1 as the core genes for osteomyelitis and DFU (Fig. 8I). The former was mainly involved in the regulation of immunity, while the latter played an important role in the formation of extracellular matrix.

## 4. Discussion

Chronic osteomyelitis increases the incidence of type 2 diabetes mellitus in humans and mice. Inflammation, mental illness, and lack of exercise are risk factors for the development of DFU in patients with osteomyelitis.^[14]^ Patient history, including metabolic and mental health, should be closely considered when planning future treatment.

The inflammatory environment of DFU can favor the development of osteomyelitis. Studies have shown that multiple inflammatory and immune markers have been detected in patients with DFU, with elevated serum ESR, CRP, IL-6, IL-8, and decreased CCL2.^[15]^ Patients with DFU have an inflammatory state in the lesion, which can also induce peripheral neuropathy and promote the spread of infected adjacent soft tissues to deeper bone tissue, providing a favorable microenvironment for the development of osteomyelitis.^[16]^ Previous studies have reported that DFU are associated with osteomyelitis through leukocyte abnormalities, including altered numbers of leukocytes, reduced chemotactic and secretory functions, as well as increased leukocyte-associated pro-inflammatory cytokines IL-1B and IL6 and upregulation of neutrophil chemotactic factor IL8 secretion.^[17–19]^

Pro-inflammatory cytokines such as IL1B and IL-6 are considered to be significant risk factors for osteomyelitis. Among them, IL1B, IL-6, IL8, and MMPs may be involved in the common pathogenesis of silver DFU and osteomyelitis. Patients with osteomyelitis had higher levels of leukocytes, CRP, TNF-α, and IL-6.^[14]^ Neutrophils overproduce IL-1β and reactive oxygen species, leading to spontaneous inflammation of bones and surrounding soft tissues.^[19]^

Diabetic mice have increased IL-17, and iNOS expression, inflammatory macrophage and slower wound healing.^[20]^ Diabetes-enhanced IL-17 induces increased neutrophil recruitment, IL-6 and RANKL, and bone resorption, making them more pathogenic.^[21]^

There may also be overlapping pathogenesis of osteomyelitis and DFU. Immune and inflammation-related cytokines and chemokines such as IL8, IL1β, and IL17 are associated with immunomodulatory pathways in both diseases. Debridement and anti-infection are critical ways to treat osteomyelitis combined with DFU. The main focus of this study was to identify DEGs common to both osteomyelitis and DFU. Thus, identifying potential targets may provide new ideas for the treatment of osteomyelitis combined with DFU.

We screened 109 overlapping DEGs in osteomyelitis and DFU, 12 of which were core genes, including CXCL9, CXCL10, CXCL13, CXCR2, FCGR3B, IL1B, IL8, LCN2, MMP1, MMP3, MMP9, and S100A. The 12 Results of GO and KEGG functional enrichment analysis demonstrated that these core genes are primarily enriched in immune and inflammatory pathways. CXCL10 and MMP1 were further validated as hub genes using the LASSO and SVM-RFE algorithms. Cytokines and chemokines play a notable role in the pathogenesis of both diseases, such as neutrophils, IL8, IL-27, IL23, MMPs, and serum TNF, which play a key role in the pathogenesis of osteomyelitis and DFU.

According to GO and KEGG pathway analysis, the LPS signaling pathway is critical in both diseases. Osteomyelitis is usually caused by infection with Gram-negative bacteria represented by Staphylococcus aureus, and osteoclastogenesis induced by the bacterial product LPS, which promotes bone destruction in osteomyelitis.^[22]^ In osteomyelitis, LPS stimulates the MAPK signaling pathway, resulting in reduced expression of the anti-inflammatory cytokine IL-10 and reduced quantities of IL-10 recruited.^[23]^ In DFU, LPS is a significant contributor to insulin resistance and inflammation due to immunopathy and the production of oxidative stress, which directly contribute to the development of DFU.^[24]^ In addition, LPS promotes the release of inflammatory cytokines and chemokines associated with osteomyelitis, which activate NF-κB, upregulating toll-like receptor 3 (TLR3) gene expression, monocyte chemotactic protein 1 (CCL2), matrix metalloproteinase 9 (MMP9). Further, it promotes IL-8, and IL-10 gene expression and secretion.

We also identified 10 TFs that regulate the expression of these genes. Upon further validation, we identified 3 TFs highly expressed in osteomyelitis and DFU, including CRBPB, ETS2, and STAT3. Collectively, they regulated the expression of the 4 central genes (CXCL10, IL1B, MMP1, and MMP9).

C-X-C motif chemokine ligand 10 (CXCL10) is a member of the chemokine family. In osteomyelitis, T cells are the primary expressing cells of CXCL10. T cells are activated to induce CXCR3 expression, being mainly attracted to the site of inflammation where IFN-γ promotes the expression of T cell chemokine CXCL10.^[25]^ In the local microenvironment of osteomyelitis stimulated by pro-inflammatory conditions such as IL-1β, IL6, LPS, and staphylococcus aureus, the pro-inflammatory chemokine CXCL10 actively participates in the inflammatory response and can attract immune cells to the site of inflammation.^[26]^ In DFU, we discovered that CXCL10 is associated with the induction of critical pro-inflammatory cytokines (IL-1β, TNF-α, and CCL2) and activation of the NF-κB pathway.^[27]^ T cell-1 infiltrating into the DFU produces CXCL10 and pro-inflammatory cytokines such as IL-1β, TNF-α, and CCL2. Therefore, CXCL10 production in DFU may aggravate inflammation at the site of injury.^[28]^

IL1B (IL-1β) is predominantly produced by the precursor cells of the innate immune system, such as monocytes and macrophages. IL1β acts as a primary pro-inflammatory cytokine and is involved in various autoimmune inflammatory responses and cellular activities, including cell proliferation, differentiation, and apoptosis. In osteomyelitis, increased levels of IL1β expression act cohesively with IL1-α to increase NLRP3 inflammatory vesicles and immune and inflammatory responses through multiple downstream mechanisms.^[29]^ Bacterial infection delays diabetic wound healing and rapidly promotes the expression of the pro-inflammatory gene IL1β in the wound, which tends to be involved in the regulation of IL-6 and TNF-α and the expression of the vascular adhesion molecule ICAM1.^[30]^

Matrix metalloproteinase 1 (MMP1) is the first recognized protein in the family of MMPs. MMP1 has been demonstrated to play a vital role in bone extracellular matrix remodeling. Osteoblasts increase MMP1 expression under the regulation of IL-1 alpha, that the frequency of MMP1 (-1607 2G/2G) genotype in OM patients exhibits, resulting in increased levels of MMP1 2G allele in OM patients, and further promoting local healing of osteomyelitis.^[31]^ In addition, it has also been discovered that MMP1 can contribute to a reduced risk of osteomyelitis.^[32]^ In wound healing of DFU, a variety of cells, including fibroblasts, basal cells, and vascular endothelial cells, express MMP1. MMP1 has collagenase activity, which promotes the migration of related cells and proteins, and increases the percentage of type III and IV collagen to facilitate wound repair.^[33]^ Furthermore, MMP1 can contribute to the removal of necrotic tissue, promote cellular directional mobility,^[34]^ increase phagocytosis of anti-inflammatory cells, and enhance the cleansing ability of inflamed areas.^[35]^

Matrix metalloproteinase 9 (MMP9), which also belongs to the matrix metalloproteinase family, has gelatinase activity that degrades extracellular matrix and basement membrane components, with detrimental effects on endothelial integrity and collagen fibers.^[36]^ Gene expression levels of MMP9 are significantly higher in osteomyelitis than in normal subjects.^[37]^ The NF-κB signaling pathway is involved in the common pathogenesis of osteomyelitis and DFU, and MMP9 is related to pro-inflammatory cytokines and osteoclast-associated genes. Inhibition of MMP9 can suppress the LPS-induced NF-κB signaling pathway, reducing osteoclast formation and expression of pro-inflammatory cytokines and slowing down the development of osteomyelitis.^[38]^

In osteomyelitis, infections can increase the activity of MMP9 and the release of various pro-inflammatory factors such as IL1β, which in turn worsens the degree of inflammation and promotes the progression of osteomyelitis.^[39]^ Studies have shown that MMP9 is highly expressed in neutrophils of DFU, particularly in intravascular and peripheral tissues at the skin lesions of DFU. This indicates that MMP9 may be critical in the vascular remodeling of DFU.^[40]^ In DFU, MMP9 also upregulates gene levels of IL1b and CXCL1 in vascular endothelial cells (VECs), which contributes to improved interaction between VECs and leukocytes.^[39]^ Previous studies have reported that abnormal neutrophil function secretes reactive oxygen species (ROS). These can activate nuclear factor κ b (NF-κB), severely upregulating MMP9, an excess of which is harmful, and can reduce tumor growth factor (TGF)-β1 and vascular endothelial growth factor (VEGF) expression, decrease angiogenesis, and affect extracellular matrix, and wound healing.^[1]^

Other reports have examined immune and inflammatory genes associated with osteomyelitis and DFU.^[41]^ A prior study by Peter discovered that IL-1β, IFN-γ, and TNF-α were the major proinflammatory signals linking osteomyelitis and DFU using a bioinformatic approach to analyze osteomyelitis and DFU. More importantly, IL-1β, IFN-γ, and TNF-α were both elevated in osteomyelitis sera and their receptors in DFU. In diabetic ulcers, IFN-γ and TNF-α stimulate local endothelial cells in the wound and promote the secretion of T-cell and monocyte chemokines and adhesion molecules while disrupting endothelial barrier integrity.^[42]^ Their findings suggest that IL-1β, IFN-γ, and TNF-α act cohesively and may provide an important proinflammatory link between osteomyelitis and DFU.^[43]^

Due to the strong genetic similarities between osteomyelitis and DFU, in contrast to the aims of previous studies, our work focuses more on exploring TFs of hub genes and regulatory genes common to osteomyelitis and DFU. We established a complex network of protein interactions to further screen key pivotal genes by common DEGs. This integrative bioinformatics approach has been widely used in the analysis of multiple diseases and is considered to be reliable.^[44,45]^ We further analyzed TFs regulating pivotal genes and determined their gene expression levels in the original dataset.

Although core genes related to osteomyelitis^[46]^ and DFU^[47]^ have been analyzed in separate studies, no prior studies have examined the common pathogenic link between the 2 using a bioinformatics approach. Due to the high co-morbidity rate between osteomyelitis and DFU, we analyzed and identified for the first time common DEGs, pathogenic pathways, core genes, and TFs between the 2, contributing to an in-depth elucidation of the mechanisms of osteomyelitis and DFU. However, there are notable areas where our study can progress and be improved. Foremost, as a retrospective study, our study requires further external validation to support our findings. Secondly, the core genes and pathogenesis need to be further validated in an in vitro model, which will be an area of focus for potential future work.

To conclude, this is the first study to research the biomarkers and pathways of osteomyelitis and DFU using the bioinformatics tool. Our study will provide a potential direction for the pathogenesis of osteomyelitis and DFU.

## 5. Conclusions

We obtained common DEGs for osteomyelitis and DFU and performed functional enrichment analysis and further PPI network analysis. We discovered multiple common differential core genes and pathogenic mechanisms in osteomyelitis and DFU, and these differential core genes primarily determine these mechanisms. Ultimately, this study provides new possibilities to investigate further the molecular mechanisms of osteomyelitis combined with DFU.

## Author contributions

Conceptualization: Pan Fan.

Data curation: Huanhuan Ye.

Formal analysis: Pan Fan.

Investigation: Pan Fan, Huanhuan Ye.

Methodology: Pan Fan, Huanhuan Ye.

Project administration: Pan Fan, Hu Xie.

Resources: Pan Fan, Huanhuan Ye.

Software: Chenhua Zhu, Huanhuan Ye.

Supervision: Chenhua Zhu, Huanhuan Ye.

Validation: Hu Xie.

Visualization: Hu Xie.

Writing – original draft: Hu Xie.

Writing – review & editing: Hu Xie..
